# Among the Colleges

**Published:** 1897-08

**Authors:** 


### AMONG THE COLLEGES.

THE faculty of the Kansas City Veterinary College have made a few changes: George A. Johnson, D.V.M., succeeds Dr. Thomas J. Turner, Control and Eradication of Contagious Diseases; Leslie J. Allen, V. S., succeeds L. Enos Day as Demonstrator of Anatomy; James H. Cock, D.V.S., succeeds C. G. Atherton, D.V.S., as Demonstrator of Histology and Pathology; E. J. Lutz, M.D., Director of Histological and Pathological Laboratories, succeeds Thomas G. Proctor, M.D.; Alphonso L. Hunt, M.D., succeeds George Q. Smith, Ph.D., Demonstrator of Chemistry. The following new names have been added to the teaching staff: Edward P. Schaffter, V. S., Anatomy; W. A. Heck, D.V.M., Physical Diagnosis; R. P. Steddom, V.S., Veterinary Obstetrics, and Hon. Albert A. Dean, National Quarantine.

**Chairman Williams will find this an opportunity for rest after his State Board Examination.**

The National Quarantine Course will give a thorough knowledge of national quarantine laws, and this subject will be covered in a course of six months.

The fees, which have been heretofore $100, are reduced, making first year $80; second year, $80, and third year, $90. The post-graduate course is $40 instead of $50.

The following text-books are added: Canine Practice, Glass's Müller, and Ophthalmology, Van Mater.

The Faculty of the New York State Veterinary College have added the name of Edward Lockhart More, B.S., Demonstrator of Veterinary Anatomy. Darwin Abbot Morton, B.S., succeeds William Ridgely Orndorff as Instructor in Organic Chemistry. Emile Mounin Chamot's name has been dropped from the list of instructors. Fred. Douglass Smith, Instructor in Analytical Chemistry, and Blin Sill Cushman, Instructor in Quantitative Chemical Analysis, have been added to the list of instructors.

**Efforts will be made to have a conference of State Examining Boards at Nashville.**

A society of comparative medicine has been organized for the purpose of giving mutual aid in gaining general and special medical knowledge, facility in conducting the exercises of the meetings, and in presenting papers and discussions in a clear and forcible manner before an audience. A fee of $5.00 is required of each student receiving a degree. Two demonstrators in anatomy are appointed each year at a salary of $125. These positions are open to members of the graduating class and to graduates of this college who have shown special proficiency in anatomy. Students who have been one or more years in the college are eligible to appointment as demonstrators.

The Governing Faculty of the American Veterinary College have dropped the name of Charles A. Doremus, M.D., Ph.D. In the Adjunct Faculty, H. B. Ferguson, Ph.G., Phar.D., has been added on. Harry W. Bellman, D.V.S., succeeds Henry Wilson Peter, D.V.S., as House Surgeon, also Roger Twombly succeeds T. Thomas as Assistant House Surgeon.

The following text-books have been added to the list: Chemistry, Bartley's; Toxicology, Robert Friedburg; Surgical Pathology, Wagner; Ophthalmology, Van Mater; Dictionary, Keating; and Botany, Rusby and Jelliffe's Essentials.

The Harvard University have added to their list of instructors, Patrick J. Cronon, M.D.V., Instructor and Assistant Surgeon at Free Clinic. Tuition fees of special students, laboratory course alone, or with other courses, $150 a year. Elective full course, $45; for a half course, $25 a year. Any special student who attends a course of instruction for only a part of the year must pay the full year's fee for the course, except that a student who is liable for the fee of $150 a year is entitled to the same remissions as undergraduates.

The McGill University have added to their list of examiners the names of J. A. Coutre and A. W. Clement. The following text book has been added : *Diseases of the Dog*, by George Müller, translated by A. Glass, V.S.

The Educational Convention at Milwaukee in July discussed the all-important resolution : *Resolved*, That the State should exercise supervision over degree-conferring colleges through some properly constituted tribunal having power to fix a minimum standard of requirements for admission to or graduation from such institutions, and with the right to deprive of the degree-conferring power such institutions not conforming to the standard so prescribed.

Commissioner of Education Harris, referring to the importance of this question, said there were over four hundred degree-conferring colleges in the United States. He thought forty would meet all the requirements. This is a step in the right direction, and there can be none with greater interest in this matter than the veterinary profession, and there should be much interest displayed by the Association of Faculties in helping to arrive at some conclusions that would hasten the accomplishment of the ends arrived at. The boards of veterinary medical examiners in the several States will all bear uniform testimony to the need of this as well as a uniform degree.

McKILLIP VETERINARY COLLEGE.

Faculty: Leo. Thurliman, M.S., Ph.G., Professor of Chemistry and Physics, has succeeded Otto Folin, B.S. E. M. Broecken, V.M.D., Professor of Histology and Microtechnology, has relinquished his connection, and F. S. Schoenleber, M.S.A., D.V.S. Professor of Anatomy and Histology, and I. D. Rawlings, M.S. M.D., Professor of Bacteriology, have been added to fill his place.

Practising Staff: John B. Boomer, M.D.V., Assistant Surgeon and Pharmacist, has succeeded F. C. Leith, M.D.C.

**We want to hear more from the association of faculties—referred to Secretary Gill.**

Professional visits show a decrease of 321 ; surgical operations show an increase of 141 ; dental operations show an increase of 165 ; hospital patients show a decrease of 210 ; hospital patients, less than five days, decrease 795. Total cases treated during 1896 show a decrease of 900 from 1895; total cases treated at hospital during 1896 show a decrease of 829 from 1895.

The bacteriological laboratory is under the direction of I. D. Rawlings, M.S., M.D., Professor of Bacteriology at the Northwestern University Medical School. The instruction in the dissecting-room is conducted by the professor of anatomy, a junior prosector, and a senior demonstrator. The following will conduct the instruction for '97–'98: F. S. Schoenleber, M.S.A., D.V.S., Professor of Anatomy; Fred E. Jones, Junior Prosector, and Henry J. Washburn, Senior Demonstrator.

**Wont some-one spring a paper on the Convention—"The Veterinarian in Politics."**

Examinations: Failure in three branches will disqualify a student for graduation.

Laboratory fee has been dropped, therefore making the expense first year $80, second year $75, third year $85.

Officers: Clarence Davis has succeeded Ernest H. Brown as Vice-President; also Clyde S. Hess has succeeded Will S. Powell as Recording Secretary and Treasurer of the College Association.

### INDIANA VETERINARY COLLEGE.

E. H. Pritchard, V.S., has succeeded T. L. Armstrong as President. H. E. Zimmer, Ph.G., elected Vice-President, and S. E. Crose, A.M., M.D., have been added to the Board. H. R. Macaulay, V.S., and J. T. McShane, M.D., have relinquished their connection as trustees. T. L. Armstrong, D.V.S., J. W. Klotz, V.S., have been added to the Board.

E. H. Pritchard, V.S., has succeeded T. L. Armstrong as Meat and Milk Inspector, and John F. Geis, M.D., has succeeded W. S. Tomlin as Professor of Physiology, and John E. Pritchard, M.D., as Professor of Histology and Microscopic Pathology, will be identified with the Faculty in the coming college year.

Lecturers: Reginald W. Garstang, M.D., has succeeded Jas. E. Dedmon as instructor in General Pathology and Morbid Anatomy. F. C. Becker has succeeded John E. Pritchard in Bacteriology. H. Smock, V.S., has succeeded E. H. Pritchard on Hygienic Breeding and General Management of Domestic Animals. The name of J. T. McShane, M.D., on Microscopic Pathology has been dropped from the list of lecturers.

Much stress is laid on clinical instruction for the coming year.

The college curriculum will be changed to a three-years' course after the session of '97–'98.

Alumni Association have elected the following officers: President, Thos. Gaddes, M.D., V.S.; Vice-President, Joe Creedon, V.S.; Secretary, Louis A. Greiner, V.S.; Treasurer, Ferd. A. Mueller, Ph.G., V.S.; Executive Committee, Samuel Rodybaugh, Charles Gause, Harry Smock, Wilbert Ramsey, and J. B. Heaton.

The objects of this Association are to promote and perpetuate the sentiments of general brotherhood among the graduates, to advance the interests and extend the influence of the Indiana Veterinary College and to encourage a high standard of veterinary education.

